# Mobility hinders malaria elimination goals in the Brazilian Amazon

**DOI:** 10.1371/journal.pcbi.1014843

**Published:** 2026-10-06

**Authors:** Nicholas J. Arisco, Pablo S. Fontoura, Cassio Peterka, Marcia C. Castro

**Affiliations:** 1 Center for Environmental Studies, Williams College, Williamstown, Massachusetts, United States of America; 2 National Malaria Prevention and Control Program – Ministry of Health of Brazil, Brasília, Distrito Federal, Brazil; 3 Secretaria de Vigilância em Saúde e Ambiente, Ministério da Saúde, Brasília, Distrito Federal, Brazil; 4 Department of Global Health and Population, Harvard T.H. Chan School of Public Health, Boston, Massachusetts, United States of America; University of Washington, UNITED STATES OF AMERICA

## Abstract

**Introduction:**

Human mobility across the Brazilian Amazon—shaped by frontier development, land-use change, mining activity, and climatic shocks—remains a major barrier to malaria elimination. Brazil aims to eliminate *Plasmodium falciparum (Pf)* by 2030 and *Plasmodium vivax (Pv)* by 2035, yet malaria incidence has plateaued since 2020. We quantify parasite-specific mobility and assess how importations drive malaria reintroduction and reestablishment in areas approaching elimination.

**Methods:**

We analyzed 5,521,411 de-identified malaria cases from Sivep-Malaria (2003–2023) across 808 municipalities in the nine-state Brazilian Amazon. We (i) used multivariable monthly sequence analysis to classify “Municipal Malaria Importation Trajectories” (MMITs) using local/imported composition, incidence per 1,000, and source/sink network typologies; (ii) characterized mobility networks by parasite species and locality type (rural, urban, settlement, indigenous, mining); and (iii) developed a parasite-specific rule-based classification procedure to identify reintroduction, and defined reestablishment as sustained local transmission.

**Results:**

Sequence analysis produced five MMIT clusters, spanning municipalities nearing elimination to areas with sustained high local transmission and distinct profiles of declining transmission with intense vs. moderate importation. Overall, 959,281 cases (17.38%) were imported. Cases were predominantly *Pv* (81.68%), with *Pf* comprising 17.15%; *Pf* was more common among imported than locally acquired infections. Mobility patterns shifted after mid-2020: by 2023, > 50% of exported cases originated in mining localities despite ~0.2% land coverage, with *Pf* importation increasingly concentrated in municipalities overlapping the Yanomami Indigenous Land. From 2006–2023, we identified 63 *Pf* and 221 *Pv* reintroduced locally acquired cases following ≥3 years of zero local transmission, leading to reestablishment in 6 and 23 municipalities, respectively, and generating 864 subsequent *Pf* and 6,524 subsequent *Pv* cases. Post-reintroduction transmission persisted for long periods (mean 762 days for *Pf*; 815 days for *Pv*).

**Conclusion:**

Malaria elimination in Brazil is increasingly hindered by parasite-specific mobility, especially linked to mining–indigenous confluences, and by repeated reintroduction in receptive municipalities nearing elimination (notably along the arc of deforestation). Mobility-informed surveillance and targeted reactive strategies are essential to achieve and sustain Brazil’s malaria elimination goals.

## Author summary

Malaria elimination depends not only on reducing local transmission, but also on preventing infections from being carried into places where malaria has already declined or disappeared. In the Brazilian Amazon, human movement is shaped by mining, settlement, urban care-seeking, Indigenous territories, environmental change, and frontier development. Using more than 5.5 million malaria case records from 2003–2023, we examined how infected people move between municipalities and how these movements affect Brazil’s malaria elimination goals. We found that imported malaria cases remain common and that recent mobility patterns have shifted sharply toward mining and Indigenous areas, especially in Roraima and municipalities overlapping the Yanomami Indigenous Land. Although *Plasmodium vivax* causes most malaria cases, imported *P. falciparum* infections have become increasingly concentrated in highly mobile mining-linked populations. We also identified municipalities where malaria returned after at least three years without local transmission, sometimes leading to years of renewed transmission. These findings show that elimination strategies must track both parasite species, acute land-use changes, and human mobility. Surveillance systems that identify major sources and receivers of imported infections can help Brazil target interventions where they are most needed and prevent malaria from becoming reestablished.

## Introduction

Human mobility, defined as both transient and migratory movements, is widespread in the Brazilian Amazon [1,2]. Four interconnected dynamics have historically influenced human mobility (mobility hereafter) into and throughout the region: (i) political forces and governmental programs that attracted business and farmers [2], (ii) socioeconomic opportunities [1], (iii) environmental degradation, infrastructure, and land-use change [3,4], and (iv) extreme climatic events and seasonal weather that result in population displacement (mostly transient) [3,5]. Modern frontier expansion and mass human movement into the Amazon began in the mid-1960s, spurred by a military dictatorship bent on development of the region for national security concerns and extraction of resources [6]. These state-sponsored development initiatives, including roads, agribusiness expansion, mineral extraction, logging, and human settlement led to rapid population growth and a transformation of the southern and eastern Amazonian landscapes [7,8]. Negative externalities to human health arose from the environmental degradation inherent to these activities; conditions became more favorable for malaria vectors while simultaneously exposing high-risk, lower-income settler populations to a paucity of healthcare and protection against diseases [9]. As a result, malaria surged along the Amazon Frontier [10,11]. This cycle of mobility, environmental change, and exposure to malaria persists today, and is worsened by extreme climatic events, such as severe droughts, which are becoming more frequent and severe due to climate change and can render rivers unnavigable, cutting off riverine communities from essential goods, services, and healthcare [5].

Brazil aims to achieve malaria elimination and zero malaria deaths by 2035 in distinct phases, beginning with the elimination of *Plasmodium falciparum (Pf)* (17.8% of the 2024 malaria burden in Brazil) by 2030, and ending with elimination of *Plasmodium vivax (Pv)* (80.4% of the 2024 burden) by 2035 [12]. Since 2020, the number of cases has stalled around 140 thousand. In 2024, the number of cases was just 1.17% lower than the annual cases recorded in 2023. Mobility of malaria-infected individuals is a well-known barrier to elimination, not just in Brazil, but globally [13,14]. Core to the mechanism by which mobility challenges malaria elimination are the concepts of receptivity and vulnerability. Receptivity is defined as the ability of an ecosystem to sustain malaria transmission, including the presence and favorable conditions for proliferation (climate and appropriate breeding habitat) of *Nyssorhynchus* mosquitoes, the presence of human populations, and the degree to which resources to prevent malaria exist in the region [15]. Vulnerability is determined by importation rates and is defined as the likelihood that infectious individuals or vectors will enter a region, modulated by the receptivity of the region to promote subsequent disease transmission [15]. Undisturbed forest systems in the Amazon are not densely populated by *Nyssorhynchus darlingi*, but when deforestation [11,16] and land-use changes [17] occur, these newly modified landscapes are highly receptive to malaria transmission. Climatic conditions, including microclimatic variability as a function of land-use changes [18], interact with the land system further altering receptivity. These dynamics are related to mobility patterns, including altering major sources and sinks of mobile, malaria-infected individuals, which have shifted geographically over time but are continually dominated by within-state, shorter distance movements [19,20].

Concerningly, malaria cases in the northern Brazilian state of Roraima increased 110% between 2017 and 2021, where illegal mining has ravaged the Yanomami indigenous lands [17,21]. Illegal miners (*garimpeiros*) move throughout the area via boat and air transport, and commonly travel to the nearby city of Boa Vista, Roraima’s capital, for care for malaria infections [22,23] Furthermore, malaria cases in Brazil are spatially concentrated; roughly 20 municipalities held 80% of malaria cases in 2023, and 52.8% of all cases originated in indigenous or mining (*garimpo*) areas [23]. Though malaria is decreasing across most of the Brazilian Amazon, cases in these areas have been rapidly rising, and have undergone novel ecological changes that may modulate receptivity as seen in neighboring countries [24]. Further complicating the scenario is a markedly higher and increasing *Pf* infection abundance among mobile *garimpeiros*, which may lead to rapid distribution of the parasite to areas with historically low transmission levels [25]. The advent of rapid expansion of *garimpo* activity in indigenous lands represent a dramatic spatial, demographic, and ecological shift of the malaria playing field compared to years as recent as 2003–2015, where malaria declined in traditional frontier areas. This presents a challenge to be met with new methods to reduce malaria burden in Brazil, with emphasis on the use of surveillance as a tool for elimination at the parasite-specific level including mobility patterns [12,26]. Fortunately, Brazil has a national epidemiological surveillance system of notified malaria cases that can be leveraged to estimate mobility and other case details for individuals who receive care for malaria from Brazil’s vast network of government-run testing facilities [27].

In the face of the challenges that mobility poses to Brazil’s malaria elimination goals, we leverage this data source to comprehensively analyze mobility in the Brazilian Amazon at the parasite-specific level between 2003 and 2023. First, we classify municipalities by trends in mobility over time using sequence analysis and create “Municipal Malaria Importation Trajectories” (MMITs). Second, we identify municipalities as major sources and sinks of *Pv* and/or *Pf* cases. Finally, we highlight regions where malaria elimination is particularly challenged due to the reintroduction of transmission following imported cases. We estimate the number of cases that originated from those reintroductions and the duration that transmission persists after reintroduction. Our results provide actionable insights to guide Brazil’s final steps toward malaria elimination, enabling the development of mobility-focused, targeted surveillance and intervention strategies at the subnational level.

## Methods

### Study area

We limited our analysis to municipalities (n = 808) in the nine states that compose the Brazilian Amazon: Rondônia (RO), Acre (AC), Amazonas (AM), Roraima (RR), Pará (PA), Amapá (AP), Tocantins (TO), Maranhão (MA), and Mato Grosso (MT). All maps are based on 2022 (the most recently updated) municipality boundaries. The boundaries of three new municipalities were drawn in Brazil and during the duration of the dataset: Mojuí dos Campos, PA (created in 2013); Ipiranga do Norte, MT (created in 2005); and Itanhangá, MT (created in 2005). For the years that these municipalities did not exist, their values were marked as missing.

### Data and case definitions

We obtained de-identified malaria case data at the individual-level, spanning the years 2003–2023, via collaboration with the National Malaria Prevention and Control Program (NMPCP). We used malaria data from the Malaria Epidemiological Surveillance Information System (Sivep-Malaria), which contains all tests (including negative results) and cases that were notified and received care.

Each registered test in Sivep-Malaria is accompanied by a broad suite of epidemiological information. We defined a positive malaria case as any test in which the result is positive. We excluded from our analysis cases labeled as relapses as well as any cases originating outside of Brazil. Malaria cases in Brazil are diagnosed most often via blood smear microscopy, followed by rapid diagnostic tests (RDT); a small number are confirmed via polymerase chain reaction (PCR). Malaria cases were classified by the parasite species causing the infection. We primarily considered *Pv* and *Pf*. *Plasmodium ovale* (*Po*), *P. malariae* (*Pm*) and mixed infections (*Pf*/*Pv*, *Pf*/*Pm*) were dealt with depending on the analysis (details listed below). If cases were classified in Sivep-Malaria as “Not *falciparum*”, we assumed these to be *Pv*.

Sivep-Malaria records include key variables for identifying imported malaria cases and details about those cases: the probable municipality of infection (determined via travel history and symptom onset at the time of diagnosis), the municipality of notification (where the individual received care), the date of symptom onset, the date of notification, and for both infection and notification locations, the classification type of each locality (rural, settlement, urban, indigenous, or mining) pre-designated by the NMPCP and included in the dataset. To calculate mobility, we followed the methods employed by Arisco et al. [27], in which the municipality of infection serves as the start point, and municipality of notification serves as the end point of a directional path. We defined an imported case as any in which these municipalities differ, and further classify imported cases as within- or between-states. We stratified these cases by parasite species, and infection and notification locality types. The final dataset included 5,521,411 malaria cases across 808 municipalities between 2003 and 2023. In 587 instances (0.01% of all cases), the municipality of infection was missing; in 83,149 instances (1.51%), the locality of infection was missing; and in 5,231 instances (0.09%), the locality of notification was missing. Observations with missing values were removed only from analyses requiring the missing field. For example, locality-specific analyses only used data in which we knew the locality of notification and/or infection, whereas in municipal-level analyses, including the sequence analysis and reintroduction/reestablishment classification procedure, we did not need locality data and thus included all but 587 observations, or 0.01% of total cases.

### Sequence analysis

We performed multichannel sequence analysis to classify and compare municipal-level malaria importation trajectories (MMITs) at the monthly level across the Brazilian Amazon from January 2003 to December 2023. Sequence analysis is a data-driven method for comparing longitudinal trajectories based on the timing, ordering, and persistence of states over time [28]. We selected this approach because our primary objective was exploratory: to characterize and map heterogeneous importation patterns across a large spatial extent and long study period, rather than estimate the mechanistic processes underlying malaria transmission. In this analysis, each municipality was represented by ordered monthly sequences across four categorical state variables: the ratio of locally acquired to imported cases, malaria incidence, source typology, and sink typology.

To handle the three municipalities that were missing for several months across the study period, we used transition-rate substitution costs and set the substitution cost for transitions from the missing state to zero so that mismatches driven solely by missing months did not inflate pairwise distance. This preserves temporal alignment and prevents the three municipalities with brief gaps in the beginning of the time series from appearing spuriously dissimilar. These pre-creation periods were treated as missing rather than historically reconstructed from parent municipalities and cannot be mapped onto historical municipal boundaries.

Four variables were incorporated into our analysis: (i) the ratio of locally acquired to imported cases; (ii) the monthly municipality malaria cases per 1,000 mid-year population (based on the municipality of infection); (iii) the “source” type of the municipality; and (iv) the “sink” type of the municipality. For variable (i), we calculated the number of locally acquired and imported cases, assigning each municipality-month to one of six possible categories: all imported, mostly imported (>50%), equal, mostly locally acquired (>50%), all locally acquired, and no cases. Because sequence analysis requires trajectories to be represented as ordered sequences of discrete states, we classified each municipality-month (variable ii) into five mutually exclusive incidence classes of cases per 1,000 mid-year population: zero, >0 and <1, ≥1 and <10, ≥10 and <50, and ≥50. While the selected cut points were chosen to match values used in the Brazilian NMPCP, MMIT classifications may be sensitive to alternative threshold definitions.

For variables (iii) and (iv), we classified municipality-months by source or sink typologies via network analysis. For each municipality and month, we first calculated two “sink” (receiver) metrics: sink strength (the total number of malaria cases received from other municipalities) and sink degree (the number of distinct municipalities from which imported cases were received). Similarly, we computed two “source” (exporter) metrics: source strength (the total number of cases exported to other municipalities) and source degree (the number of distinct municipalities to which a municipality exported cases to). For each metric and month, we standardized the values using z-scores. Municipalities with a z-score above 2 in a given month were considered exceptionally high in that metric for that month. Municipalities were then assigned to one of five typology classes for both sink and source roles in each month: Type A: super-spreader/receiver (exceptionally high degree and strength, z > 2 for both metrics); Type B: targeted, high-volume spreader/receiver (exceptionally high strength only); Type C: diffuse, low-volume spreader/receiver (exceptionally high degree only); Type D: residual spreader/receiver (neither metric exceptionally high); not a sink/source. These states comprised variables (iii) and (iv). For further analysis, we also calculated these typologies by municipality-year, stratified by parasite species of infection only including *Pv* and *Pf* cases (excluding mixed, *Po*, and *Pm*).

To compare the temporal dynamics among municipalities, we calculated pairwise dissimilarity matrices for each sequence domain using optimal matching with transition rate-based substitution costs. We then summed individual dissimilarity matrices to create a composite dissimilarity matrix capturing differences across all variables. Hierarchical clustering was performed using Ward’s linkage (Ward D2 in R) on the composite dissimilarity matrix to group municipalities with similar MMITs. The resulting dendrogram was examined visually, and the optimal number of clusters was guided by average silhouette score and simplicity of interpretability. All sequence analysis was conducted using the TraMineR package in R version 4.5.1.

### Identifying malaria reintroduction and reestablishment

Locally acquired cases (thus eligible to be defined as reintroduction) were defined as single parasite infections (*Pf* or *Pv*), while imported cases are those that enter the municipality after a period of at least three consecutive years without any locally acquired cases (defined as the baseline) and were allowed to be mixed or single parasite infections because these cases contribute to the stock of potential parasites. This increases the probability that post-importation locally acquired cases arise from those importations, as it captures the entire parasite-specific load entering the municipality. Because the baseline requires a 3-year case-free period, municipalities are considered at risk for reintroduction classification only once they achieve this prerequisite; intervals that have not yet met the baseline are not counted toward reintroduction risk. Thus, we created reintroduction and reestablishment statistics for both *Pf* and *Pv* for the years 2006–2023.

We developed a rule-based classification procedure to summarize observed surveillance data (S1 Text) and to classify malaria cases as reintroductions in line with the World Health Organization Malaria Terminology [15]. The classification procedure identifies imported cases that occurred in a municipality after baseline, and then creates an immediate buffer period to allow for parasite transmission and incubation of 24-days for *Pv* and 30-days for *Pf*. These windows were selected to conservatively capture the expected interval between importation and observable locally acquired infection, based on typical malaria incubation periods and likely delays between symptom onset, care-seeking, and notification. When no locally acquired cases occur during this buffer period, its end marks the beginning of a 45-day reintroduction window. If, after the buffer period, new local transmission was detected in the reintroduction window, these locally acquired cases were defined as importation-driven reintroduction events and characterized as first-generation (F1) locally acquired cases. If locally acquired cases occurred after the reintroduction window, we characterized these as second-generation (F2) locally acquired cases. This chain of first- and second-generation locally acquired cases was considered as malaria reestablishment if three or more locally acquired cases were observed for three consecutive years. We also assessed whether, after reestablishment, another period of at least three consecutive years without any locally acquired cases was observed. We calculated the transmission duration for each reintroduction episode as the number of days between the initial reintroduction event and the last subsequent locally acquired case before this three-year interruption. Durations are treated as time-to-clearance outcomes and are right-censored when a three-year zero-case interval is not observed before the end of the study period on December 31, 2023. Municipalities that never return to zero within the observation window are retained as ongoing chains: we still compute the elapsed duration from the initial reintroduction to the last observed locally acquired case. Under the primary definition, a two-year zero-case interval, for example, is not sufficient to declare clearance; such gaps remain part of the same transmission chain until a full three-year zero-case period is observed. The classification procedure and calculations were run in R version 4.5.1.

## Results

### Municipal malaria importation trajectories (MMITs)

Our sequence analysis resulted in five unique clusters of municipalities based on the four monthly variables used in our analysis (Fig 1). The average silhouette score for five clusters was 0.74. We characterize these clusters as: I - municipalities with declining local transmission and very intense mobility; II - municipalities with declining local transmission and less intense mobility; III - municipalities with sustained high local transmission and minimal importation; IV - municipalities with on-going low levels of local transmission and stable importation; V - municipalities nearing elimination.

**Fig 1:**
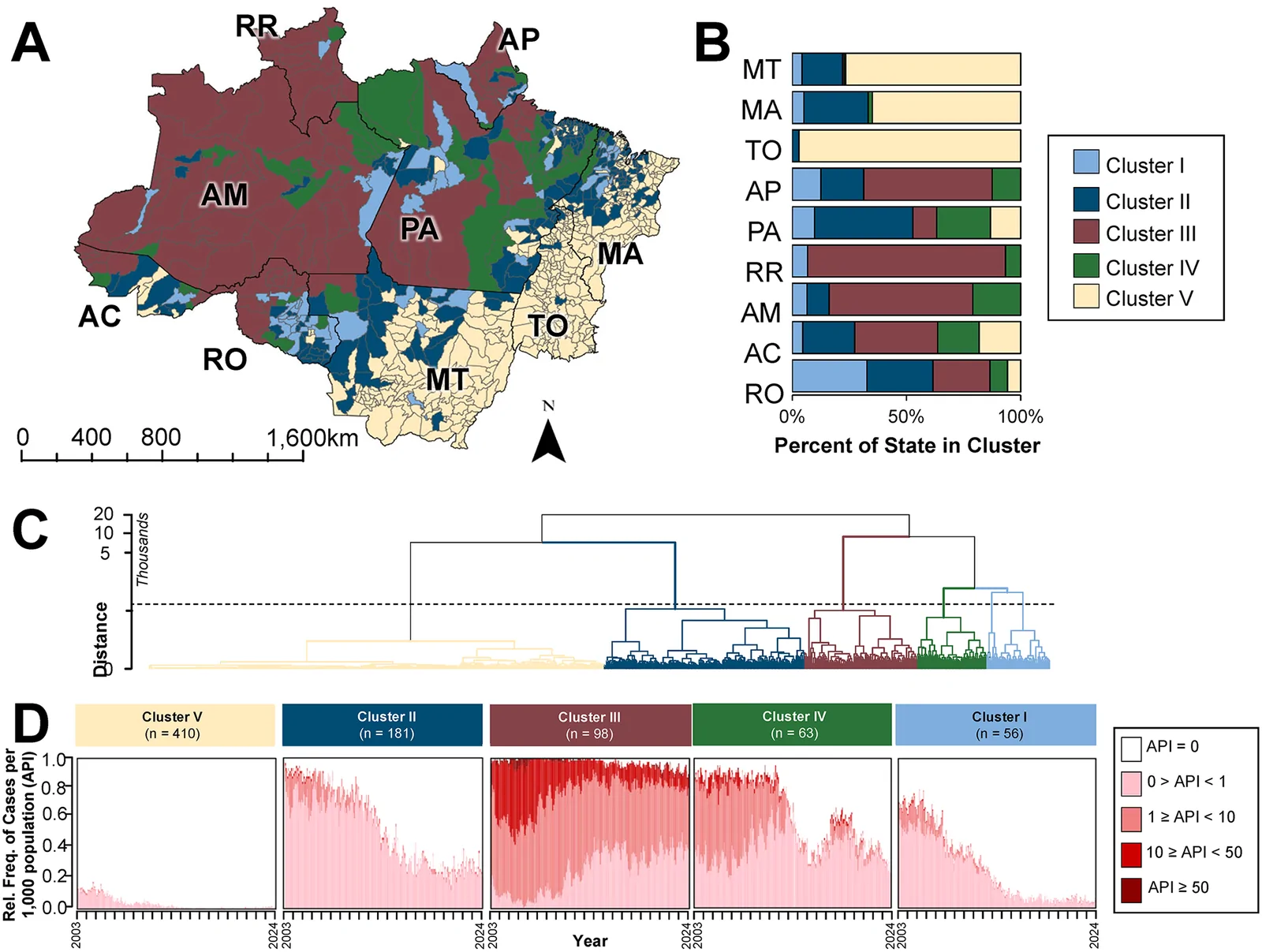
Municipal Malaria Importation Trajectories (MMITs). **A**. Map of cluster typology based on hierarchal clustering sequence analysis of mobility characteristics of municipalities at monthly intervals. State abbreviations: RO: Rondônia, AC: Acre, AM: Amazonas, RR: Roraima, PA: Pará, AP: Amapá, TO: Tocantins, MA: Maranhão, MT: Mato Grosso. **B.** Percentage of municipalities in each Amazonian state classified into each type of cluster. **C**. Dendrogram with dashed line indicating clustering height cutoff (clusters estimated using Ward D linkage algorithm). **D**. Distribution plots for each cluster considering the API variable included in the clustering algorithm. Basemap accessed at Natural Earth (Public Domain), https://www.naturalearthdata.com/about/terms-of-use/.

We classify more than half of the municipalities in the Amazon as nearing elimination (cluster V); 93.7% of these are in the states of Tocantins, Maranhão, and Mato Grosso, along the historical Amazonian arc of deforestation. Though locally acquired cases have declined to near zero, low-level importation remains in these areas. Municipalities with MMITs most similar to those nearing elimination are in cluster II, which in recent years demonstrated stable but low transmission with most cases being imported. These municipalities are geographically positioned between the historical arc of deforestation, and a region of more intense malaria transmission characterized by cluster III, which spreads across 98 municipalities predominantly in Roraima, Amazonas, northern Rondônia, northern Amapá, and western Pará. Mobility is common across municipalities in cluster III, though most cases are locally acquired.

Two additional MMITs were identified. Cluster I includes municipalities with declining local transmission but with a sustained, intense importation of malaria cases. Super-receivers are highly represented in this MMIT as opposed to others, and imported cases dominate the epidemiological profile of malaria cases. These municipalities tend not to export as many malaria cases as they receive. Cluster IV presented the highest level of similarity to cluster III (intense, local transmission). This MMIT includes 63 municipalities distributed throughout the Amazon. Temporal trends indicate that these areas have not experienced the marked declines seen in clusters V and II, but also do not exhibit the high, sustained transmission characteristic of cluster III.

### Mobility networks stratified by parasite species and locality type

Of the 5,521,411 malaria cases notified in the Brazilian Amazon from 2003 to 2023, 959,281 (17.38% of total) were imported. There were 946,628 *Pf* cases (17.15% of total cases; 19.05% of imported cases), 4,509,538 *Pv* cases (81.68% of total cases; 79.24% of imported cases), 62,720 mixed infections (1.13% of total cases; 1.65% of imported cases), 1,889 *Pm* cases (0.03% of total cases; 0.05% of imported cases), and 49 *Po* cases. Total malaria cases have remained largely stable since 2018, holding steady in the range of 128,633–152,918 per year, while *Pf* cases reached a 10-year high in 2021 of 21,581 cases, *Pv* reached a 10-year low in 2022 of 108,315 cases, and mixed *Pv*/*Pf* infections reached a 10-year high in 2023 of 2,806 cases. During the study period, malaria cases were 14% more likely to be *Pf* if the case was imported compared to if it was locally acquired; this same statistic was 55% for mixed infections.

Mobility patterns varied spatiotemporally based on parasite species and locality where infection and notification occurred. Between 2003 and 2021, most malaria cases originated in rural localities (Fig 2). However, beginning in mid-2020, there was a notable shift, with a growing proportion of cases originating in mining and indigenous localities. By 2023, over 50% of exported malaria cases originated in mining localities, despite these comprising only 0.2% of the total land area in the Brazilian Amazon, with the majority emerging from the Yanomami indigenous land. On the one hand, malaria cases in mining localities made up nearly half of exported cases from 2021-2023 but constituted <5% of locally acquired cases. On the other hand, locally acquired cases mostly occurred in indigenous localities. These mining and indigenous localities substantially overlap with the 26 municipalities responsible for 80% of all malaria cases reported in 2023.

**Fig 2:**
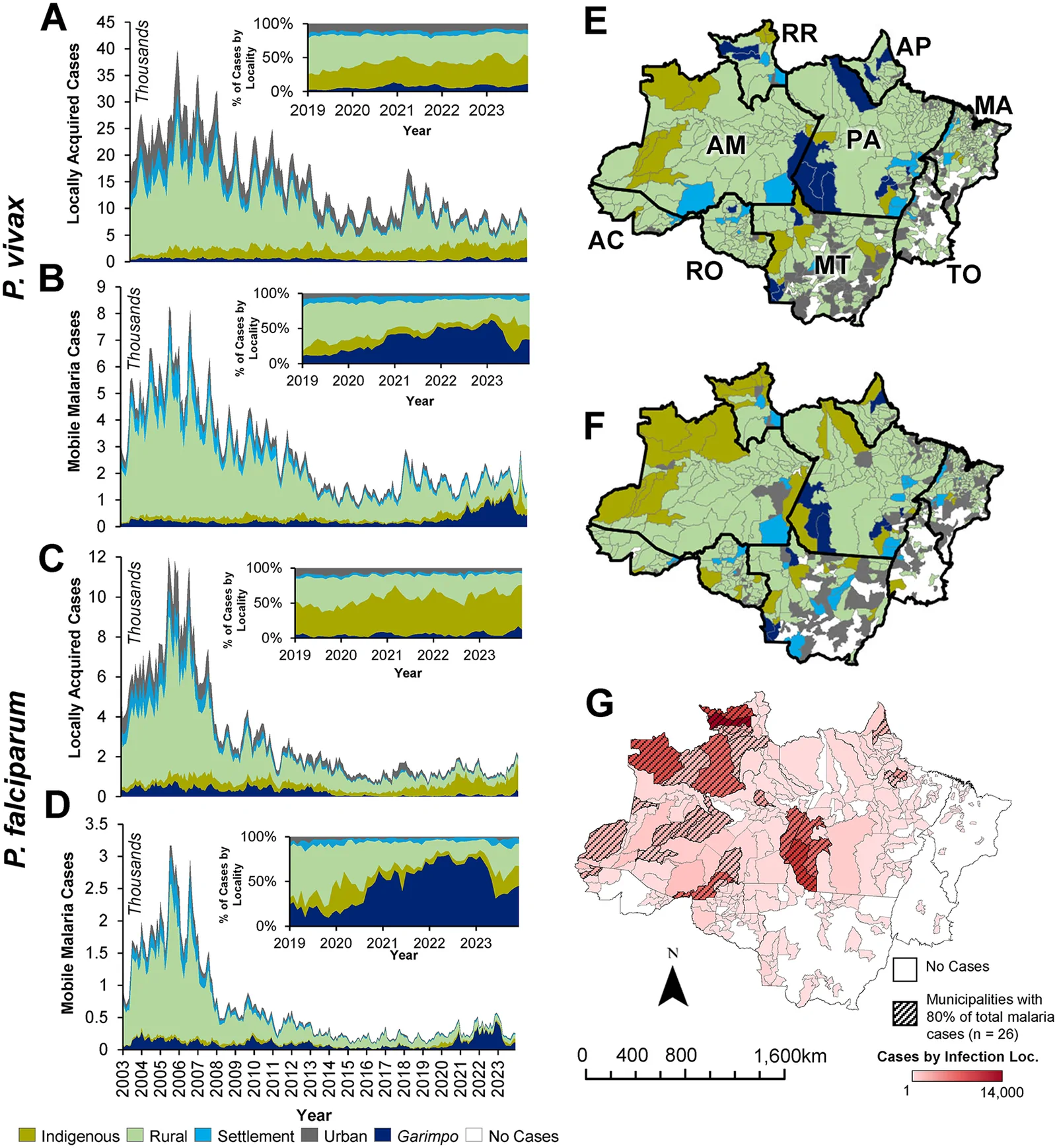
Malaria cases by parasite, mobility, and locality of infection. **A.** *Pv* area plot indicating the monthly time series of locally acquired malaria cases and **B.** mobile malaria cases by infection locality, colored by locality type. **C.** *Pf* area plot indicating the monthly time series of locally acquired malaria cases and **D.** mobile malaria cases by infection locality, colored by locality type. Cutouts show the most recent five years of data, specifically as percentage each state comprises of the total cases in each category. **E.** Most common locality type in municipality in which mobile malaria cases originated and **F.** in which locally acquired cases originated between 2003 and 2023, colored by locality type of infection. **G.** Total malaria cases in 2003 by municipality of infection, with municipalities comprising 80% of malaria cases highlighted. State abbreviations: RO: Rondônia, AC: Acre, AM: Amazonas, RR: Roraima, PA: Pará, AP: Amapá, TO: Tocantins, MA: Maranhão, MT: Mato Grosso. Basemap accessed at Natural Earth (Public Domain), https://www.naturalearthdata.com/about/terms-of-use/.

Imported *Pv* cases have remained relatively stable since 2017, whereas imported *Pf* cases steadily increased through 2023 before declining by the end of the year. This rise in *Pf* case importation was concentrated in mining localities in Roraima state, particularly in municipalities overlapping the Yanomami indigenous land. Up to half of all imported *Pv* cases originated in mining or indigenous localities, while this number reached 75% among imported *Pf* cases. Among locally acquired cases, most occurred in indigenous localities by the end of 2023 regardless of parasite. Locally acquired *Pf* cases originated more frequently in indigenous localities than did locally acquired *Pv* cases. Locally acquired cases in indigenous localities demonstrated no seasonality, contrary to rural localities which experienced regular, sub-annual, sinusoidal seasonality. Of note, very few malaria cases had a common notification and infection location in mining localities, regardless of parasite species, though many infections occurred in mining localities with notification in non-mining (typically urban) localities.

Considering municipal-level typologies of malaria importation by parasite and by year, 19 municipalities were *Pf* super-spreaders and 25 were *Pv* super-spreaders between 2003 and 2023 (Fig 3). Super-spreaders of both *Pf* and *Pv* became less common over time, and Porto Velho and Itaituba most frequently were super-spreader municipalities across the study period. In the most recent four years, Alto Alegre and Mucajaí (Roraima) were the only *Pf* super-spreaders. These municipalities, along with Itaituba (Pará), were also the only *Pv* super-spreaders between 2020–2023, and all overlap indigenous lands with active mining operations. Super-receivers were largely urban centers and state capitals; Porto Velho (Rondônia’s capital), Macapá (Amapá’s capital), Boa Vista (Roraima’s capital), and Manaus (Amazonas’ capital) were in the top five for both *Pf* and *Pv*. Super-receivers were also more consistent over time and space, particularly for *Pv* infections.

**Fig 3:**
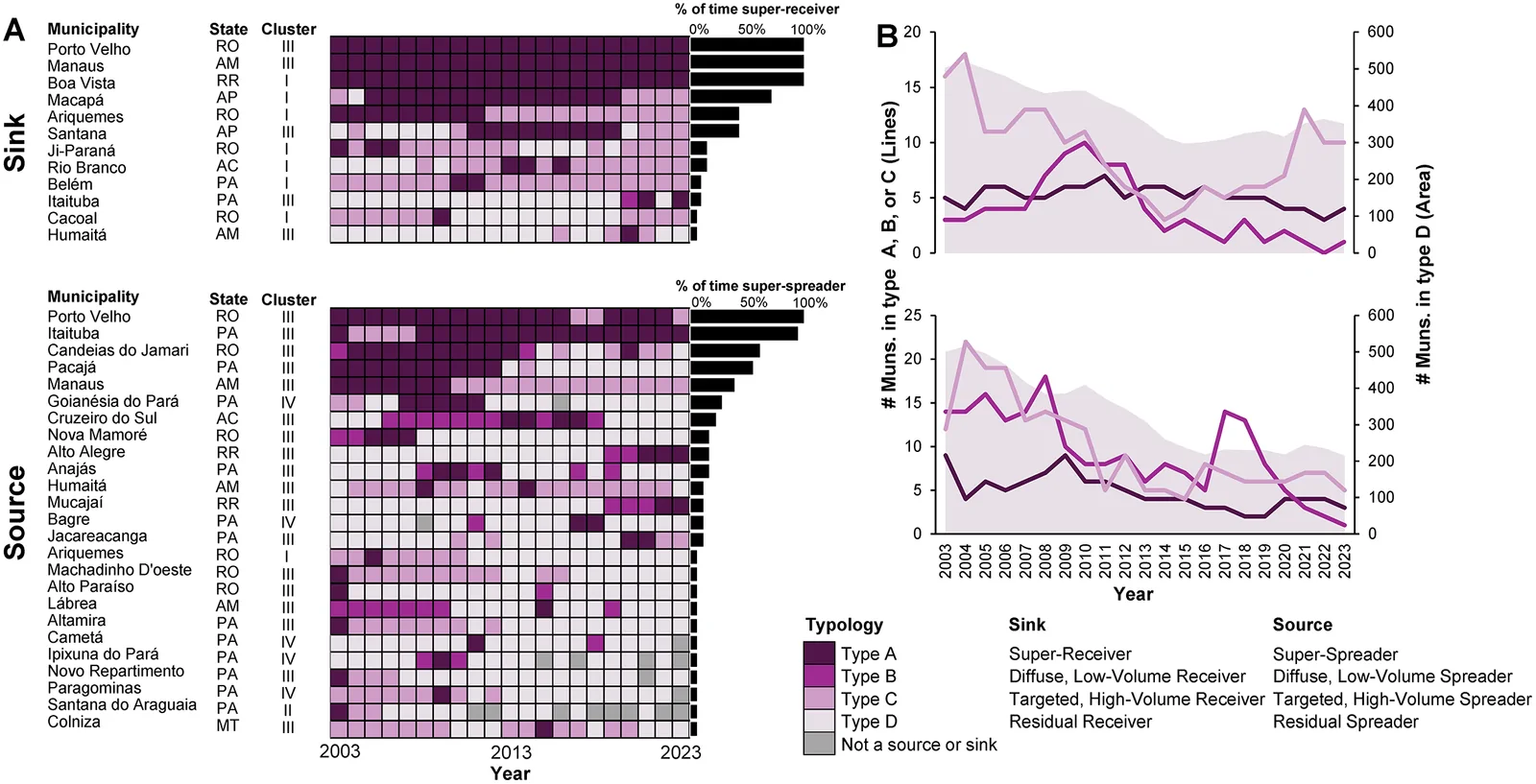
Analysis of source/sink typology for *Pv* (*Pf* in S1 Fig). **A.** Heatmaps of annual typology for municipalities categorized as Type A (super-spreaders or super-receivers) between 2003-2023 and percentage of years the municipality remained in Type A over the study period. **B.** Line graphs show the number of municipalities in each source/sink typology by year. Numbers for typologies A, B, and C are represented in the Y-axis, and for typology D (the most common, indicated as a shaded area) in the secondary Y-axis. State abbreviations: RO: Rondônia, AC: Acre, AM: Amazonas, RR: Roraima, PA: Pará, AP: Amapá, TO: Tocantins, MA: Maranhão, MT: Mato Grosso.

### Reintroduction and reestablishment of malaria transmission by parasite species

We present a conceptual diagram for assessing reintroduction by parasite species (Fig 4). This diagram represents a workflow upon which surveillance systems might be able to establish reintroduction with appropriate data, including areas of infection and notification, and parasite species of infection. We find that between 2006 and 2023, 63 *Pf* and 221 *Pv* locally acquired cases were reintroduced into municipalities that had experienced zero malaria cases for three or more years. *Pf* reintroductions were distributed across 33 municipalities in eight states, with 73% occurring in the states of Pará and Maranhão. *Pv* reintroductions were distributed across 113 municipalities in nine states, with 80% occurring in Pará, Maranhão, and Mato Grosso. These *Pv* and *Pf* reintroductions resulted in malaria reestablishment in 138 unique municipalities (76% of which were in Pará, Maranhão, and Mato Grosso) for a total of 864 subsequent *Pf* cases and 6,524 *Pv* cases.

**Fig 4:**
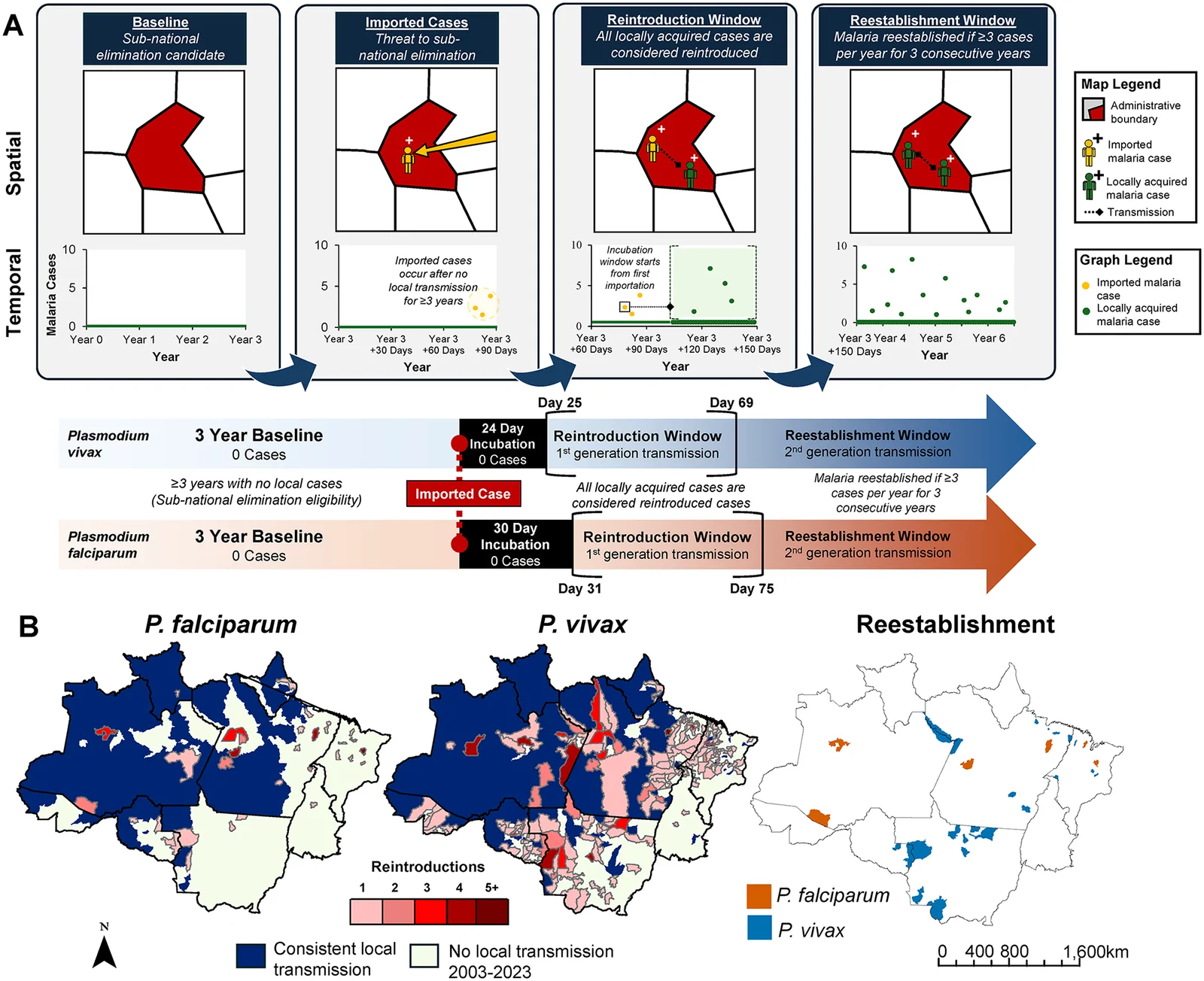
Malaria reintroduction and reestablishment in the Amazon, 2006-2023. **A.** Diagram of reintroduction classification procedure, specific to *Pf* and *Pv* (Supplement). **B.** Spatial distribution of reintroduction and reestablishment events by parasite, areas with ongoing local transmission, and areas that have recorded no parasite-specific locally acquired cases between 2003-2023. Basemap accessed at Natural Earth (Public Domain), https://www.naturalearthdata.com/about/terms-of-use/.

Of the 113 municipalities with *Pv* reintroduction and 33 municipalities with *Pf* reintroduction, 23 and 6, respectively, experienced malaria reestablishment. Some municipalities experienced first- and second-generation cases after reintroduction. Of those instances, *Pf* transmission after reintroduction persisted for 762.3 days (range: 5–3,781 days; SD: 971.24 days), while *Pv* transmission after reintroduction persisted for 814.60 days (range: 4–3,910 days; SD: 849.02 days). The municipalities that experienced *Pf* reestablishment were Boca do Acre (Amazonas, 3,781 days, start date: 1/4/2013), Fonte Boa (Amazonas, 1,903 days, start date: 3/15/2014), Rurópolis (Pará, 1,861 days, start date: 3/11/2009), Pinheiro (Maranhão, 1,808 days, start date: 8/7/2007), Coroatá (Maranhão, 1,650 days, start date: 2/7/2007), and Tomé-Açu (Pará, 1,417 days, start date: 10/2/2009). Of the 23 municipalities that experienced *Pv* reestablishment, the five with the longest subsequent transmission periods were Santa Bárbara do Pará (Pará, 3,910 days, start date: 6/18/2007), Tucumã (Pará, 3,323 days, start date: 4/4/2014), Guarantã do Norte (Mato Grosso, 3,045 days, start date: 11/14/2008), Santo Antônio dos Lopes (Maranhão, 2,591 days, start date: 6/16/2009), and Gonçalves Dias (Maranhão, 2,447 days, start date: 2/11/2016).

### Case examples of reintroduction and reestablishment

We highlight three municipalities that experienced reintroduction and reestablishment during the study period: Boca do Acre (Amazonas), Cametá (Pará), and Gonçalves Dias (Maranhão) (Fig 5). Boca do Acre belongs to cluster III, which is characterized by consistent and intense local transmission. Between December 2010 and December 2013, zero locally acquired *Pf* cases were notified in this municipality. Imported cases between 11/23/2012 and 12/11/2012 were determined to have likely resulted in two reintroduced *Pf* cases on 1/4/2013 and 2/8/2013. After this, a spike in *Pf* cases ensued, leveled out in 2018, and resurged in 2019. Transmission persisted through the end of the study period. Cametá belongs to cluster IV, which is characterized by on-going low levels of local transmission and stable importation. This municipality experienced two extreme spikes in *Pv* cases following intense bouts of *Pv* importation. Prior to these importation episodes, zero locally acquired *Pv* cases were reported for at least one year (a less strict cutoff compared to other municipality examples). During the first spike in locally acquired *Pv* cases, we estimate that reintroduction resulted in 17,353 *Pv* cases between 2010 and 2013. A similar dynamic occurred during the second spike, which amounted to 11,102 *Pv* cases between 2017 and 2021, followed by a period of very few locally acquired *Pv* cases. Gonçalves Dias belongs to cluster II, which is characterized by declining, less intense mobility. Between 2007 and 2010, zero locally acquired *Pv* cases were reported in this municipality. An imported *Pv* case in the beginning of 2010 spurred a resurgence in low-level, locally acquired *Pv* transmission amounting to 21 locally acquired *Pv* cases over 87 weeks. This was followed by an eight-year period of zero locally acquired *Pv* cases, after which a locally acquired case appeared in early 2020 and transmission persisted through the end of the study period. In this case, most likely one or more asymptomatic cases were imported, not detected by regular surveillance, contributing to reintroduction and re-establishment.

**Fig 5:**
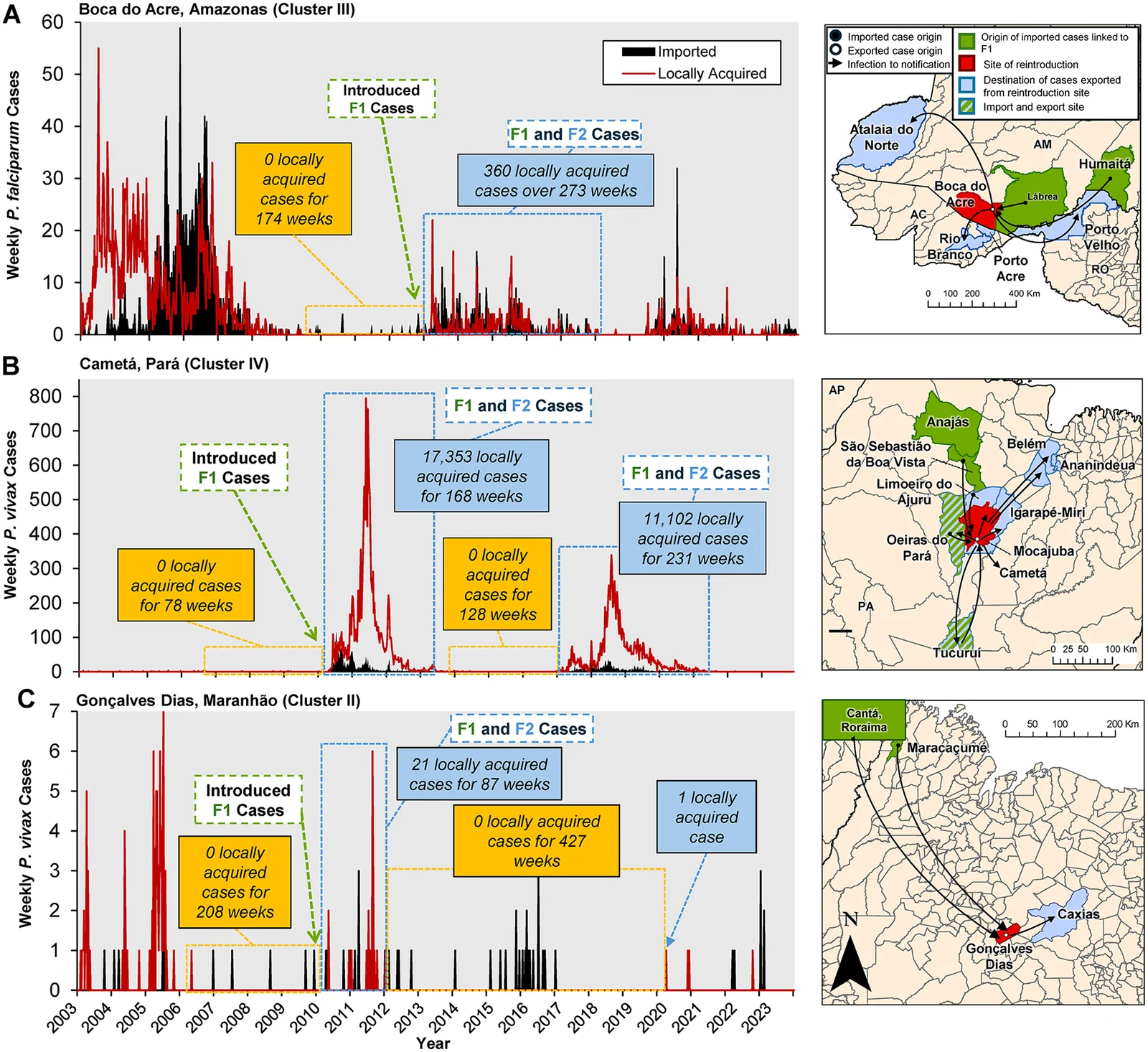
Case examples of reintroduction and reestablishment of malaria. **A.** Boca do Acre, Amazonas. *Pf* reintroduction event after more than 3 years without local transmission. The municipality was classified as Cluster III. **B.** Cametá, Pará. *Pv* reintroduction event in Cluster IV. Two reintroduction and re-establishment events occur. **C.** Gonçalves Dias, Maranhão. *Pv* reintroduction event in a municipality with historically low malaria transmission (Cluster II). Basemap accessed at Natural Earth (Public Domain), https://www.naturalearthdata.com/about/terms-of-use/.

## Discussion

We analyzed two decades of individual-level malaria case data and revealed a comprehensive picture of parasite-specific mobility across the Brazilian Amazon. We leverage a theory-informed, rule-based classification procedure for using surveillance data to identify malaria reintroduction and reestablishment at the parasite-specific level. Our results show that while *Pv* continues to account for most cases, imported *Pf* cases have risen sharply, particularly among highly mobile *garimpo* populations. In addition, mobility has led to repeated reintroductions and reestablishment of local transmission even in municipalities that had previously achieved zero parasite-specific cases for three years. Though *Pv* mobility remains high, associated reintroduction events are more widely distributed and result in larger, longer-lasting secondary outbreaks of the disease. These patterns may have contributed to a concentration of *Pv* and *Pf* infections in a small number of high-transmission municipalities that act as persistent super-spreaders, especially where indigenous lands and mining areas overlap.

These findings present a two-fold challenge to Brazil’s malaria elimination goals: (1) malaria cases, and particularly *Pf* infections, which are the first target of elimination efforts, have been disproportionately consolidating and increasing in hard-to-reach and highly mobile populations, and (2) areas that are vulnerable to malaria, and which are trending toward subnational elimination, can regress to prolonged reestablishment from imported malaria cases.

The first challenge is represented by a fundamental shift in the malaria epidemiological landscape of Brazil since 2017. *Garimpo* areas—representing less than 0.2% of the Amazon’s land—now account for over 50% of mobile malaria cases and an even larger share of mobile *Pf* infections. Our sequence analysis demonstrates that these areas have experienced consistently high malaria transmission and mobility since 2003 and are epidemiologically most dissimilar to municipalities closer to elimination. These municipalities, for example Alto Alegre, Mucajaí, and Itaituba, tend to follow the MMIT characterized by consistent, intense, local transmission, are super-spreaders, and may have outsized significance for national elimination prospects. Between 2021 and 2023, Boa Vista was the only *Pf* super-receiver, and the bordering municipalities of Alto Alegre and Mucajaí were the only *Pf* super-spreaders. This presents a key example of a localized area for Brazil to engage in active surveillance of sub-populations that may amount to outsized progress toward the 2030 *Pf* elimination goal. Innovative tools such as Malakit—self-diagnosis and self-treatment kits distributed in French Guiana and parts of the Brazilian Amazon—have shown promise and particular efficacy in reducing the *Pf* burden among *garimpeiros* in the Guiana Shield [29,30]. Their success has been attributable to their alignment with the needs of highly mobile and hard-to-reach mining populations: they reduce reliance on fixed health facilities, enable earlier diagnosis and treatment in remote settings, and are paired with user training.

Yet, reducing the capacity to engage in *garimpo* activity is also a necessary step in addressing this challenge. Brazilian President Lula da Silva has escalated anti-mining enforcement, slowing the expansion of new mines in the Yanomami Indigenous Land [31]. However, the epidemiological effects of these enforcement actions remain uncertain. Malaria cases in Indigenous areas of Roraima remained high through 2024 before declining in 2025–18,505 cases, while data for 2026 are not yet available (Sivep-Malaria). Importantly, there is limited empirical evidence on whether, how quickly, or to what extent *garimpeiro* populations left affected areas following enforcement actions. Thus, reductions in visible mining activity should not be assumed to translate immediately into equivalent reductions in mobility-related malaria risk. Several factors may underlie persistent transmission: Yanomami communities may be continually threatened by a highly fragmented and modified landscape rife with vector habitat from abandoned mines; communities may be experiencing long-term health consequences related to undernutrition [32], attenuating immune systems and leading to more severe malaria [33]; healthcare systems may still be recovering from the shock of *garimpo* operations; and part of the increase could reflect better coverage in diagnosis [23].

The second challenge is geographically distinct from the first challenge and is focused primarily along the arc of deforestation, the broad crescent-shaped frontier of forest loss along the southern and eastern edges of the Brazilian Amazon, extending roughly from Maranhão and Pará through Mato Grosso and Rondônia into Acre, where roads, settlement, agriculture, ranching, logging, and mining have historically concentrated deforestation [7]. Though the extent of locally acquired malaria transmission along this region has shrunk between 2004 and 2022, a “ring” of mostly imported malaria cases has persisted and become more intense [20]. Despite this region largely following an MMIT representative of nearing elimination, we demonstrate here that it remains both receptive and vulnerable to malaria reintroduction and re-establishment. If subnational malaria elimination [34] were to become a core component of the Brazilian malaria elimination plan, these municipalities may see an outsized benefit from heightened active surveillance. Such a method of surveillance as a tool for malaria elimination is in line with a core pillar of the WHO Global Technical Strategy for Malaria 2016–2030 [26].

Further, the reintroduction classification procedure we develop here may serve as a useful framework for implementing more efficient reactive case detection (RACD) strategies in these municipalities [35]. More concretely, municipalities repeatedly identified as super-spreaders or super-receivers could be prioritized for enhanced surveillance at key transit points linking mining areas to urban centers, including river ports, airstrips, and high-volume testing facilities. Source–sink pairings identified through the mobility network could also support inter-municipal notification protocols, so that imported cases diagnosed in urban centers trigger timely investigation, testing, or RACD in the likely municipality of infection. Certain municipalities in Brazil may be in the subnational elimination endgame, yet resurgence of malaria from imported cases remains a threat. Understanding common mobility networks, demographic profiles of imported malaria cases, and how these may increase over time can provide RACD programs the ability to stratify resources more effectively [36]. Furthermore, ecological change in the Brazilian Amazon rapidly alters the malaria playing field via the creation of vector habitat and frontier malaria dynamics [16] and may contribute to the source and sink tendencies of a municipality, as well as the receptivity and vulnerability of the area. This work also highlights the value of robust public health surveillance infrastructure. Our ability to characterize malaria mobility, source/sink patterns, and reintroduction dynamics depends on the consistent recording of case origin, notification location, parasite species, and timing in Sivep-Malaria. Detailed longitudinal surveillance systems such as this are essential for translating routine case reporting into actionable elimination strategies. Understanding the ecology, epidemiology, health systems, and demography of municipalities that have experienced malaria reestablishment, versus those that have avoided it in the face of imported malaria cases, will be a crucial step in achieving and maintaining malaria elimination in Brazil.

This paper has several limitations. First, the MMIT classification should be interpreted in light of the discretization required for sequence analysis. Because incidence and network measures were converted into categorical monthly states, the resulting clusters may be sensitive to the selected cut points. Approaches that retain continuous longitudinal information may yield alternative, complimentary classifications. Second, we excluded cases originating outside Brazil in order to focus on within-country municipal mobility. As a result, our estimates capture internal importation rather than total importation pressure, and likely underestimate vulnerability in border municipalities, particularly in Roraima where malaria dynamics are strongly linked to cross-border movement with Venezuela [19]. Third, the 3-year zero-local-transmission baseline used to define reintroduction is intentionally conservative. While this increases confidence that classified events represent true reintroduction after interruption of parasite-specific local transmission, it may miss outbreaks following shorter 1- or 2-year gaps. Our estimates should therefore be interpreted as a conservative subset of detected reintroduction and reestablishment events. Fourth, probable municipality of infection is based on patient-reported travel history and may be misclassified, particularly among mobile *garimpo* populations who travel regularly and may be less likely to report illegal activity. This could underestimate rural or mining sources and overstate the apparent role of urban notification centers as sinks. Finally, Sivep-Malaria captures detected infections and may miss asymptomatic infections. Asymptomatic mobile carriers could introduce parasites without appearing as imported cases, so some transmission chains may only be detected after later symptomatic infections. Our estimates should therefore be interpreted as detected mobility, reintroduction, and reestablishment events.

## Conclusion

Human mobility remains a significant barrier to malaria elimination in Brazil, enabling repeated reintroduction and reestablishment of the disease. Gaining a deep understanding of the interplay between receptivity and vulnerability—two pillars often discussed in elimination but rarely measured at scale—will be critical for Brazil to achieve malaria elimination by its intended goal of 2035. As hotspots for mobility shift and malaria cases consolidate around distinct ecological transitions and demographic groups, surveillance must be able to nimbly track and treat malaria cases while minimizing the infectious period of individuals. To secure lasting elimination, Brazil will need to enhance surveillance for mobile populations and invest in real-time indicators of receptivity alongside predictive modeling of vulnerability linked to migration, ecological change, and economic activity.

## Supporting information

S1 Text Formal logic for rules-based classification procedure for classification of malaria reintroduction, re-establishment, and transmission duration (*P. falciparum and P. vivax*). (PDF)

S1 Fig Analysis of source/sink typology for *Pf.* A. Heatmaps of annual typology for municipalities categorized as Type A (super-spreaders or super-receivers) between 2003–2023 and percentage of years the municipality remained in Type A over the study period. **B.** Line graphs show the number of municipalities in each source/sink typology by year. Numbers for typologies A, B, and C are represented in the Y-axis, and for typology D (the most common, indicated as a shaded area) in the secondary Y-axis. State abbreviations: RO: Rondônia, AC: Acre, AM: Amazonas, RR: Roraima, PA: Pará, AP: Amapá, TO: Tocantins, MA: Maranhão, MT: Mato Grosso.
(TIF)
