# Supplementary material for: Mobility hinders malaria elimination goals in the Brazilian Amazon
Source: PLoS Comput Biol. 2026 Oct 6;22(10):e1014843. doi: 10.1371/journal.pcbi.1014843 (PMC13641346; doi:10.1371/journal.pcbi.1014843)
Supplement: Supplementary file 1 [file pcbi.1014843.s001.pdf]

Mobility Hinders Malaria Elimination Goals in the Brazilian Amazon

Nicholas J. Arisco; Pablo S. Fontoura; Cassio Peterka; Marcia C. Castro

Supplementary Text S1

Formal rules-based classification procedure for classification of malaria reintroduction, re-establishment, and transmission duration (*P. falciparum* and *P. vivax*)

S1.1 Overview

We formalize a rules-based classification procedure, aligned with WHO Malaria Terminology, to classify parasite-specific reintroduction events and ensuing local transmission in Brazilian municipalities. Locally acquired cases are restricted to single-species infections (*Pf* or *Pv*); imported cases may be single-species or mixed and contribute to the species-specific import "stock." Detection proceeds via (i) verification of a three-year baseline with no local cases, (ii) a species-specific buffer period with no local cases, (iii) a fixed reintroduction window in which the first local case defines an importation-driven reintroduction time, (iv) construction of the subsequent local chain (F1/F2) until a gap > three years, (v) assessment of re-establishment, and (vi) computation of the episode duration.

S1.2 Notation and data

Let $\mathcal{M}$ be the set of municipalities and $\mathcal{P} = \{\mathrm{Pf}, \mathrm{Pv}\}$ the parasite species. Time is indexed in days $t \in \mathbb{T}$ (Gregorian). For $m \in \mathcal{M}$, $p \in \mathcal{P}$:

$$\begin{aligned} L_{m,p}(t) \in \mathbb{N}_0 &\ : \text{local (autochthonous) \textit{single-species} cases of species } p \text{ with notification date } t, \\ I_{m,p}(t) \in \mathbb{N}_0 &\ : \text{imported cases whose recorded species set includes } p \text{ (single or mixed), date } t. \end{aligned}$$

Species-specific constants:

$$\begin{aligned} b_{\mathrm{Pv}} &= 24 \text{ days}, \qquad b_{\mathrm{Pf}} = 30 \text{ days} && \text{(buffer lengths)}, \\ W &= 45 \text{ days} && \text{(reintroduction window length)}, \\ H &= 1095 \text{ days} && \text{(baseline and chain gap horizon of 3 years)}. \end{aligned}$$

For calendar-year summaries, let $Y(y) = \{t : t \text{ falls in year } y\}$ and

$$C_{m,p}(y) = \sum_{t \in Y(y)} L_{m,p}(t).$$

Species mapping from SIVEP-Malaria. Let each line-listed case c carry a SIVEP species label set $S(c) \subseteq \mathcal{P}$. We aggregate to daily counts by municipality m and date t as

$$\begin{aligned} L_{m,p}(t) &= \sum_{c \in \mathcal{L}_m(t)} \mathbf{1}\{S(c) = \{p\}\}, \\ I_{m,p}(t) &= \sum_{c \in \mathcal{I}_m(t)} \mathbf{1}\{p \in S(c)\}, \end{aligned}$$

where $\mathcal{L}_m(t)$ and $\mathcal{I}_m(t)$ are the sets of local and imported cases (respectively) with notification date t in m, and $\mathbf{1}\{\cdot\}$ is the indicator.

S1.3 Baseline and eligible imports

Define the baseline indicator

$$B_{m,p}(t) = \mathbf{1}\left\{ \sum_{u=t-H}^{t-1} L_{m,p}(u) = 0 \right\}. \tag{1}$$

An import on day t_0 is *eligible* if $I_{m,p}(t_0) > 0$ and $B_{m,p}(t_0) = 1$. Let

$$\begin{aligned} \mathcal{T}^{\text{imp}}_{m,p} = \Big\{ t_0 \in \mathbb{T} \,: I_{m,p}(t_0) > 0,\ B_{m,p}(t_0) = 1, \\ \sum_{u=t_0}^{t_0+b_p} L_{m,p}(u) = 0 \text{ (buffer free of local)} \Big\}. \end{aligned} \tag{2}$$

The three-year baseline implies that, given data beginning in 2003, reintroduction ascertainment is reliable from 2006 through 2023.

S1.4 Buffer, window, and reintroduction time

For $t_0 \in \mathcal{T}^{\text{imp}}_{m,p}$, define the buffer and window

$$\begin{aligned} \mathbb{B}_p(t_0) &= [t_0,\, t_0 + b_p], \\ \mathbb{W}_p(t_0) &= (t_0 + b_p,\, t_0 + b_p + W]. \end{aligned}$$

Let the union of all species-p reintroduction windows be

$$\mathcal{U}_{m,p} \;=\; \bigcup_{t_0 \in \mathcal{T}^{\text{imp}}_{m,p}} \mathbb{W}_p(t_0). \tag{3}$$

We declare a reintroduction when the first local case occurs inside $\mathcal{U}_{m,p}$:

$$\tau^*_{m,p} \;=\; \inf\{\tau \in \mathcal{U}_{m,p} :\, L_{m,p}(\tau) > 0\}, \qquad R_{m,p}(\tau^*_{m,p}) = 1, \tag{4}$$

with the convention that $\inf \emptyset$ is undefined (no reintroduction). Definition (3) resolves overlapping windows by earliest-time precedence.

S1.5 Local chain (F1/F2) and termination

Given a reintroduction at $\tau^* = \tau^*_{m,p}$, we define the chain of local transmission dates $\mathcal{F}_{m,p}(\tau^*) = \{t_0, t_1, \ldots, t_K\}$ recursively by

$$\begin{aligned} &t_0 = \tau^*, \\ &t_k = \min\{t > t_{k-1} :\, L_{m,p}(t) > 0 \text{ and } t - t_{k-1} \le H\}, \quad k \ge 1, \end{aligned} \tag{5}$$

stopping when no t satisfies the condition. Equivalently,

$$\mathcal{F}_{m,p}(\tau^*) = \{\, t \ge \tau^* : L_{m,p}(t) > 0 \text{ and no gap } > H \text{ since the most recent local} \,\}.$$

The chain is inactive once the inter-event gap exceeds H days.

S1.6 Re-establishment criterion

Let the chain-restricted annual counts be

$$C^{\mathcal{F}}_{m,p}(y;\tau^*) \;=\; \sum_{t\in Y(y)\cap\mathcal{F}_{m,p}(\tau^*)} L_{m,p}(t). \tag{6}$$

We say re-establishment occurs for the episode begun at τ^* if there exist three consecutive years $y, y+1, y+2$ such that

$$C^{\mathcal{F}}_{m,p}(y;\tau^*) \geq 3, \qquad C^{\mathcal{F}}_{m,p}(y+1;\tau^*) \geq 3, \qquad C^{\mathcal{F}}_{m,p}(y+2;\tau^*) \geq 3. \tag{7}$$

S1.7 Transmission duration

For an episode starting at τ^* with last local date $t_K = \max \mathcal{F}_{m,p}(\tau^*)$, define the duration

$$D_{m,p}(\tau^*) \;=\; (t_K - \tau^*) + 1 \text{ days}. \tag{8}$$

If the observation window ends while the chain is active (right-censoring), report $D_{m,p}(\tau^*)$ using the last observed t_K and note the censoring.

S1.8 Logical specification (species p in municipality m)

Step 1: Baseline screening: For each day t, compute $B_{m,p}(t)$ per (1).

Step 2: Eligible imports: Collect t_0 satisfying (2); form $\mathcal{U}_{m,p}$ in (3).

Step 3: Reintroduction: If $\exists\tau \in \mathcal{U}_{m,p}$ with $L_{m,p}(\tau) > 0$, set τ^* per (4).

Step 4: F1/F2 chain: Build $\mathcal{F}_{m,p}(\tau^*)$ per (5); terminate after a gap $> H$.

Step 5: Re-establishment: Evaluate the three-year criterion on $C^{\mathcal{F}}_{m,p}(y;\tau^*)$.

Step 6: Duration: Report $D_{m,p}(\tau^*)$.

Edge cases. (i) Multiple eligible imports producing overlapping windows are handled by (3)–(4). (ii) Same-day multiplicities are collapsed in $L_{m,p}(t) \in \mathbb{N}_0$. (iii) Distinct episodes in the same municipality/species are handled by repeating Steps 2–6 after termination.

Table 1: **S1.9 Symbol table**: Mathematical symbols and their definitions used in the formulae above.

Symbol	Definition
$L_{m,p}(t)$	Local single-species cases of p at date t in m
$I_{m,p}(t)$	Imported cases whose species includes p at t in m
b_p	Species buffer: $b_{\text{Pf}} = 30$, $b_{\text{Pv}} = 24$ (days)
W	Reintroduction window length, 45 days
H	Baseline/chain gap horizon, 1095 days
$B_{m,p}(t)$	Baseline indicator (no local in prior H days)
$\mathcal{T}^{\text{imp}}_{m,p}$	Eligible import dates satisfying baseline and buffer-free
$\mathbb{W}_p(t_0)$	Window after an eligible import t_0
$\mathcal{U}_{m,p}$	Union of windows over eligible imports
$\tau^*_{m,p}$	First local date in $\mathcal{U}_{m,p}$ (reintroduction time)
$\mathcal{F}_{m,p}(\tau^*)$	Local chain (F1/F2) after τ^* until gap $> H$
$C_{m,p}(y)$	Annual local count; $C^{\mathcal{F}}_{m,p}(y;\tau^*)$: chain-restricted
$D_{m,p}(\tau^*)$	Episode duration in days

S1.10 R snippets (for reproducibility)

(a) Reintroduction finder (Pf defaults).

Listing 1: Species-specific reintroduction finder.

```
find_reintroductions <- function(df,
                                 buffer_days   = 30L,   # Pf: 30; set 24 for Pv
                                 window_days   = 45L,
                                 lookback_days = 365L*3L) {
  n <- nrow(df)
  reintro <- integer(n)
  for (i in seq_len(n)) {
    if (!is.na(df$imported[i]) && df$imported[i] > 0L) {
      start_check <- max(1L, i - lookback_days)
      if (all(is.na(df$locally_acquired[start_check:(i-1)]) |
              df$locally_acquired[start_check:(i-1)] == 0L)) {
        end_buf <- min(i + buffer_days, n)
        if (all(is.na(df$locally_acquired[i:end_buf]) |
                df$locally_acquired[i:end_buf] == 0L)) {
          start_win <- min(i + buffer_days, n) + 1L
          end_win   <- min(i + buffer_days + window_days, n)
          if (start_win <= end_win) {
            j <- which(df$locally_acquired[start_win:end_win] > 0L)[1]
            if (!is.na(j)) reintro[start_win + j - 1L] <- 1L
          }
        }
      }
    }
  }
  reintro
}
```

(b) Chain and duration.

Listing 2: F1/F2 chain and episode durations.

```
mark_chain_until_gap <- function(df, lookback_days = 365L*3L) {
  active <- integer(nrow(df)); last_local <- NA_integer_; seen <- FALSE
  for (i in seq_len(nrow(df))) {
    if (df$reintro[i] > 0L) { active[i] <- df$locally_acquired[i]; last_local <- i; seen
    <- TRUE }
    else if (seen && df$locally_acquired[i] > 0L && (is.na(last_local) || (i - last_local
    ) <= lookback_days)) {
      active[i] <- df$locally_acquired[i]; last_local <- i
    } else if (seen && !is.na(last_local) && (i - last_local) > lookback_days) break
  }
  return(active)
}

compute_durations <- function(df, lookback_days = 365L*3L) {
  starts <- as.Date(character()); durs <- integer(); n <- nrow(df); i <- 1L
  while (i <= n) {
    if (df$reintro[i] > 0L) {
      start <- df$date[i]; last <- i; j <- i + 1L
      while (j <= n) {
        if (df$locally_acquired[j] > 0L && (j - last) <= lookback_days) last <- j
        else if ((j - last) > lookback_days) break
        j <- j + 1L
      }
      starts <- c(starts, start)
      durs   <- c(durs, as.integer(df$date[last] - start) + 1L)
      i <- j
    } else {
      i <- i + 1L
    }
  }
  data.frame(Start_Date = starts, Duration_Days = durs, check.names = FALSE)
}
```

S1.11 Reporting and notes

- **Reintroduction:** report $\{\tau^*_{m,p}\}$ per municipality/species.
- **F1/F2:** report $|\mathcal{F}_{m,p}(\tau^*)|$ or total cases over the chain.
- **Re-establishment:** indicate whether the three-year (3,3,3) criterion holds and the qualifying years.
- **Duration:** summarize $D_{m,p}(\tau^*)$ across episodes (e.g., median, IQR).
- **Censoring:** flag right-censored episodes.

Reference. World Health Organization. *Malaria terminology.* WHO/HTM/GMP/2016.6 (and updates).
